# When, why and how tumour clonal diversity predicts survival

**DOI:** 10.1111/eva.13057

**Published:** 2020-07-18

**Authors:** Robert Noble, John T. Burley, Cécile Le Sueur, Michael E. Hochberg

**Affiliations:** ^1^ Department of Biosystems Science and Engineering ETH Zurich Basel Switzerland; ^2^ SIB Swiss Institute of Bioinformatics Basel Switzerland; ^3^ Department of Evolutionary Biology and Environmental Studies University of Zurich Zurich Switzerland; ^4^ Department of Ecology and Evolutionary Biology Brown University Providence RI USA; ^5^ Institute at Brown for Environment and Society Brown University Providence RI USA; ^6^ Institut des Sciences de l’Evolution University of Montpellier Montpellier France; ^7^ Santa Fe Institute NM USA; ^8^ Present address: Department of Mathematics City, University of London London UK

**Keywords:** cancer, computational model, evolutionary dynamics, evolutionary forecasting, prognostic biomarkers

## Abstract

The utility of intratumour heterogeneity as a prognostic biomarker is the subject of ongoing clinical investigation. However, the relationship between this marker and its clinical impact is mediated by an evolutionary process that is not well understood. Here, we employ a spatial computational model of tumour evolution to assess when, why and how intratumour heterogeneity can be used to forecast tumour growth rate and progression‐free survival. We identify three conditions that can lead to a positive correlation between clonal diversity and subsequent growth rate: diversity is measured early in tumour development; selective sweeps are rare; and/or tumours vary in the rate at which they acquire driver mutations. Opposite conditions typically lead to negative correlation. In cohorts of tumours with diverse evolutionary parameters, we find that clonal diversity is a reliable predictor of both growth rate and progression‐free survival. We thus offer explanations—grounded in evolutionary theory—for empirical findings in various cancers, including survival analyses reported in the recent TRACERx Renal study of clear‐cell renal cell carcinoma. Our work informs the search for new prognostic biomarkers and contributes to the development of predictive oncology.

## 1 INTRODUCTION

Despite a wealth of available genomic data, the development of reliable predictive tools in oncology remains challenging (Lipinski et al., 2016; Turajlic, Sottoriva, Graham, & Swanton, 2019; Yankeelov, Quaranta, Evans, & Rericha, 2015). Although the search for prognostic biomarkers has largely focussed on the presence or absence of particular genomic aberrations, indices of intratumour heterogeneity, which depend only on aberration frequencies, not identities, have emerged as promising alternative predictors (Alizadeh et al., 2015; Jamal‐Hanjani, Quezada, Larkin, & Swanton, 2015; Maley et al., 2017; Marusyk, Almendro, & Polyak, 2012; Polyak, 2014). Higher clonal diversity has been found to predict worse clinical outcome in Barrett's oesophagus (Maley et al., 2006; Martinez et al., 2016; Merlo et al., 2010), ovarian cancer (Schwarz et al., 2015), lung cancer (Jamal‐Hanjani et al., 2017) and breast cancer (Park, Gönen, Kim, Michor, & Polyak, 2010; Rye et al., 2018). Computational modelling of premalignant somatic evolution further indicates that genetic diversity indices can predict cancer risk more reliably than the presence or absence of particular mutations (Dhawan, Graham, & Fletcher, 2016). However, studies in kidney cancer (Turajlic et al., 2018) and across cancer types (Andor et al., 2015) have found more complicated relationships between intratumour heterogeneity and its clinical impact, which elude simple explanation. It also remains unclear whether and how intratumour heterogeneity can complement established prognostic biomarkers—such as tumour stage and grade—and other proposed ecological and evolutionary indices (Maley et al., 2017).

Interpreting intratumour heterogeneity is challenging due to the complexity of tumour evolutionary dynamics (Burrell, McGranahan, Bartek, & Swanton, 2013; Cross, Graham, & Wright, 2016; Gerlinger et al., 2012; Lipinski et al., 2016; Venkatesan & Swanton, 2016; Williams, Werner, Graham, & Sottoriva, 2016). Greater heterogeneity at the scale of tumour clonal composition may reflect higher genomic instability (Hanahan & Weinberg, 2011) and may correspond to a greater likelihood of malignant cell phenotypes being present (Greaves & Maley, 2012; Maley et al., 2006, 2017). On the other hand, clonal sweeps initiated by highly adapted clones might reduce diversity within aggressive tumours (Maley et al., 2006, 2017; Robertson‐Tessi & Anderson, 2015). The frequency of such selective sweeps in turn depends on the extent of clonal interference (Lang et al., 2013; Martens, Kostadinov, Maley, & Hallatschek, 2011) and spatial constraints (Michor, Frank, May, Iwasa, & Nowak, 2003; Noble, Burri, Kather, & Beerenwinkel, 2019), which may vary between tumour types. Potentially, clonal diversity could fail as a prognostic biomarker either because it is insufficiently variable within patient cohorts or because tumour progression is so stochastically variable as to be effectively unpredictable. Even when forecasts are possible, their accuracy is expected to depend on the number and location of cells used to assess heterogeneity. Some tumour regions will tend to harbour more clonal diversity than others, and a clone's ability to expand and contribute to growth rate is constrained by intracellular interactions, which depend on the clone's location relative to other clones and to the tumour edge.

Here, we use a computational model of solid tumour evolution to characterize the relationship between clonal diversity, subsequent tumour growth rate and progression‐free survival after treatment. We identify conditions that determine the sign and strength of correlations between these variables. We thus provide insight into the evolutionary processes underlying clinical observations. Our results contribute to establishing a theoretical foundation for predictive oncology.

## 2 METHODS

### 2.1 Computational model

We simulate invasive tumour growth and evolution using a spatial, stochastic computational model specifically designed to recapitulate the spatial structure of common acinar tumour types, such as kidney, lung and breast carcinomas. This model has previously been shown to generate a pattern of branched evolution, consistent with previous observations in various cancer types (Noble et al., 2019).

Our model represents tissue as a two‐dimensional regular grid of “demes,” corresponding to localized subpopulations of interacting cells. Initially, all demes contain normal cells, except that one deme at the centre contains a single tumour cell with a higher division rate than normal cells. Tumour cells stochastically divide, mutate, die and disperse between neighbouring demes, whereas normal cells undergo stochastic division and death only. The number of cells per deme is regulated, via negative feedback, to remain approximately constant. Specifically, we assume that the cell death rate increases from zero to a large value (100 times the initial cell division rate) when the deme population size exceeds carrying capacity *K*. Whenever a tumour cell emerges via cell division, it either remains in the same deme or, with probability d, disperses to a neighbouring deme. The value of dispersal probability d is adjusted for the value of *K*, such that, in the absence of mutation, all tumours would take a similar amount of time to reach the endpoint size of one million cells (corresponding to several years in real time). We restrict our analysis to mutations that either increase tumour cell fitness (termed driver mutations) or confer resistance to treatment. The distribution of fitness effects and the nature of epistasis in tumour evolution are only poorly understood but it is reasonable to assume that biological constraints impose diminishing returns (Wiser, Ribeck, & Lenski, 2013). Accordingly in our model, driver mutations occur at rate μ and individually increase cell division rate r by a factor of 1 + *X* × (1 – *r*/*m*), where *X* is drawn from an exponential distribution with mean value *s*, and m is an upper bound on the cell division rate. Because we set m to be much larger than the initial value of r, the combined effect of drivers is approximately multiplicative. In simulations of response to treatment, we additionally model the acquisition of resistance mutations. Treatment is applied at the endpoint size (one million cells) and immediately eliminates all cells that lack resistance mutations. The tumour's subsequent growth (if any) is tracked until it again attains the endpoint size. This choice of survival outcome takes advantage of the fact that, in a simulation study such as ours, the observation time is essentially unlimited and we can look at endpoints that would be infeasible or unethical in a real clinical trial. We use Gillespie's exact stochastic simulation algorithm (Gillespie, 1977) to ensure statistically accurate simulation of cell events. Additional features of the computational model have been previously described (Noble et al., 2019) and the code is shared in a public repository (Noble, 2019a).

### 2.2 Forecast correlations

**Figure 1:**
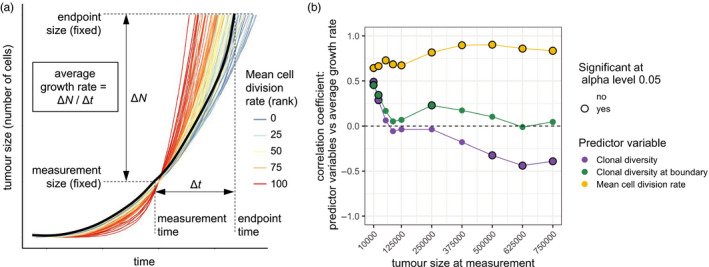
Correlations between predictor variables and future tumour growth rate for cohorts of tumours with identical parameter values. (a) Example tumour growth trajectories. In this cohort, tumours with higher mean cell division rate (red curves) typically have higher future growth rate, which results in a positive correlation coefficient. One trajectory is shown in black to illustrate the method used to calculate average growth rate. (b) Correlation coefficients between predictor variables and subsequent tumour growth rate, for different measurement sizes. Correlation coefficients that significantly differ from zero (*p* < .05) are indicated by black rings. Each point represents a cohort of 100 simulated tumours; lines are added only to guide the eye. Parameter values are *K* = 512, *μ* = 10^−5^, *s* = 0.1

We focus our study on how the principal parameters of our model—deme carrying capacity (*K*), driver mutation rate (*μ*) and mean driver fitness effect (*s*)—influence the coefficient of correlation between a predictor variable (such as clonal diversity), measured at a particular tumour size, and the rate at which the tumour grows to a larger endpoint size (Figure 1a). We define the tumour's future growth rate as.

(endpointsize‐measurementsize)/(endpointtime‐measurementtime),

where measurement size is the tumour size when the predictor variable is measured; measurement time is the corresponding tumour age; and endpoint size and endpoint time are the tumour size and age, respectively, when the tumour reaches the endpoint size. We use Spearman's rank correlation coefficient so as to allow for nonlinear correlations. Correlation coefficients closer to 1 or −1 correspond to more reliable forecasts. Analyses that instead use Kendall's τ coefficient (a concordance index) produce similar results (compare Figures 4b and S7).

### 2.3 Survival analysis

Our survival analyses use the Cox proportional hazards model. To satisfy the model's assumptions, we transform each continuous explanatory variable to obtain an approximately linear relationship with the martingale residuals (the differences between the observed numbers of progression events and the expected numbers based on the fitted null model). If a variable's effects significantly grow or diminish with time then we include a term for the interaction between that variable and log_10_(*t* + 10), where t is time measured in days (results are insensitive to the choice of the constant in this term). We conducted analyses in R using the “survival” package (Therneau, 2015) and the “survminer” package (Kassambara, Kosinski, & Biecek, 2019).

### 2.4 Clonal diversity index

We measure clonal diversity using the inverse Simpson index, defined as one divided by the sum of the squares of all clone frequencies. This index can be straightforwardly interpreted as an effective number of distinct clones. For example, if a tumour contains *n* equally abundant clones then the diversity index is 1/(*n* × (1/*n*)^2^) = *n*.

### 2.5 Clonal turnover index

Our clonal turnover index quantifies the average rate of change in clone frequencies. Specifically, for each time point t≥τ, we calculate $\Theta \left(t\right)=\sum_{i}(f_{i}\left(t\right)‐f_{i}{\left(t‐\tau \right)}^{2}$, where $f_{i}\left(t\right)$ is the frequency of clone i at time t, and τ is 10% of the total simulation time (the time from when the simulation is initiated with a single cell until the endpoint time). The clonal turnover index is then the mean value of Θ(t). Time is measured in cell generations (i.e. relative to the expected cell division time of the initial tumour clone) and the time points are evenly spaced.

### 2.6 Sampling

We variously measure clonal diversity among all cells in the simulated tumour, all cells at the tumour edge, a small random sample of cells, or one or more small localized samples of cells (simulated biopsy). We sample the edge of the tumour by selecting all demes that are directly “visible” from one of the four sides of the square lattice and, from each of these edge demes, we sample a number of cells equal to the square root of the deme carrying capacity, *K* (so the total sample size is not biased by deme size).

Biopsies are taken from small disc‐shaped regions, centred at a given proportion of the distance from the tumour's edge to its centre of mass. When four biopsies are taken from the same tumour, they are located on perpendicular transects through the tumour's centre of mass (Figure S8). The number of cells sampled from a deme is proportional to the extent of overlap between the biopsy region and the deme. If the sample size is less than the deme population size then we employ multinomial sampling of clones. Our sample size of between 100 and 4,000 cells per tumour is within the range that can be practically analysed by single‐cell sequencing.

### 2.7 Parameter values

Although cancer genomic data is broadly consistent with driver mutation rates between 10^–6^ and 10^–2^ per cell division (Bozic, Paterson, & Waclaw, 2019), most studies have settled on values close to 10^–5^ per cell division (Bozic et al., 2010; Sun et al., 2017; Waclaw et al., 2015). Likewise, we consider values of μ in the range 10^–6^–10^–4^. When we simulate cohorts of tumours with different mutation rates, we choose each *μ* value at random from a continuous uniform distribution within this range. We assume that the rate of acquiring resistance mutations is 10 times lower than the driver mutation rate, so as to simulate a treatment that is only infrequently curative.

In certain nonspatial mathematical models, driver fitness effects of less than 1% are consistent with the acquisition of multiple driver mutations during tumour growth (Bozic et al., 2010). Importantly, the same result does not necessarily hold when spatial structure and/or clonal interference inhibit the spread of beneficial mutations. Consistent with recent genomic data analysis (Williams et al., 2018), we assume the mean driver fitness effect *s* to be between 0.05 and 0.2.

We have previously found that glands of invasive, acinar tumours typically contain between a few hundred and a few thousand cells (Noble et al., 2019). We therefore consider *K* values of 64, 512 and 4,096.

Other parameter values are listed in Table 1.

**Table 1:** Parameter values used in this study

Parameter	Value(s)
Deme carrying capacity, *K*	64, 512, 4,096
Driver mutation rate, *μ*	10^−6^–10^−4^
Resistance mutation rate	*μ*/10
Mean driver fitness effect, *s*	0.05–0.2
Normal cell relative division rate	0.9
Upper bound on cell division rate, m	10
Dispersal rate	Conditional

Note

Mutation rate is measured per cell division; division rate is measured relative to the division rate of the initial tumour cell. The effect of a driver mutation with effect size s is to multiply the cell division rate r by a factor of 1 + *s*(1 – *r*/*m*), where *m* is the upper bound. Dispersal rates are set such that tumours typically take between 500 and 1,500 cell generations to grow from one to one million cells, corresponding to several years of human tumour growth.

## 3 RESULTS

### 3.1 Mean cell division rate reliably predicts future tumour growth

Before considering clonal diversity, we first look at the predictive value of mean cell division rate in our model. Cell division rate is an important component of conventional clinical grading of tumours and therefore provides a benchmark with which we can compare alternative predictor variables. To test the limits of forecastability, we examine cohorts of simulated tumours with identical parameter values and initial conditions, so that variation among growing, evolving tumours is entirely due to stochastic events.

In line with intuition, and consistent with the clinical evidence that underpins cancer staging, mean cell division rate is consistently positively correlated with future tumour growth rate in our model (Figure 1b, yellow curve; Figure S1). The correlation is however imperfect because tumour growth trajectories continue to be swayed by the stochastic accumulation of driver mutations between the measurement time and the endpoint time (the time at which the tumour reaches the endpoint size, as shown in Figure 1a). Correlations are weaker when cell division rate is measured at a very early stage of tumour growth, especially when driver mutations have only small effects on cell fitness (Figure S1). This is mostly because very small tumours typically lack sub‐clonal driver mutations and hence there is insufficient variation in cell division rate to support a correlation. When driver mutations have large fitness effects, correlations are weaker if cell division rate is measured at a very late stage (Figure S1). In general, these initial results demonstrate that, despite the effects of ongoing stochastic events, tumour growth in our models is in principle forecastable.

### 3.2 Diversity‐growth correlation depends on when and where diversity is measured

Having established potential for forecasting, we next examine correlations between clonal diversity and subsequent tumour growth rate, which we will refer to as diversity‐growth correlations. We define diversity in terms of Simpson's index (see Section 2) and define a clone as a set of tumour cells with the same combination of driver mutations (i.e. mutations that increase cell fitness). In other words, cells belong to the same clone if and only if they are identical by descent with regard to driver mutations. This definition of a clone is consistent with that used by the TRACERx Renal Consortium, which has conducted the most sophisticated clinical investigation of the association between intratumour heterogeneity and disease progression to date (Turajlic et al., 2018).

When measured at an early stage of tumour development, we find that clonal diversity is positively correlated with subsequent tumour growth rate. Conversely, clonal diversity measured at a later stage can be negatively correlated with tumour growth rate (Figure 1b, blue curve). Therefore diversity‐growth correlations importantly depend on the stage of tumour development when diversity is measured.

One factor that affects the predictive value of clonal diversity is that cells contribute unequally to tumour growth. Since our model assumes that tumours grow by invading normal tissue, the build‐up of driver mutations in the tumour core does not influence total population growth unless an especially fit clone evades clonal interference and spreads so rapidly through the tumour so as to overtake the expanding edge. As such we would predict that adaptive mutations arising near the tumour edge are more likely to increase tumour growth rate, at least over short timescales. Consistent with these considerations, negative diversity‐growth correlations are absent or less pronounced when diversity is measured only in cells sampled from the tumour edge (Figure 1b, green curve; Figure S1).

### 3.3 Diversity‐growth correlation depends on the extent of clonal turnover

**Figure 2:**
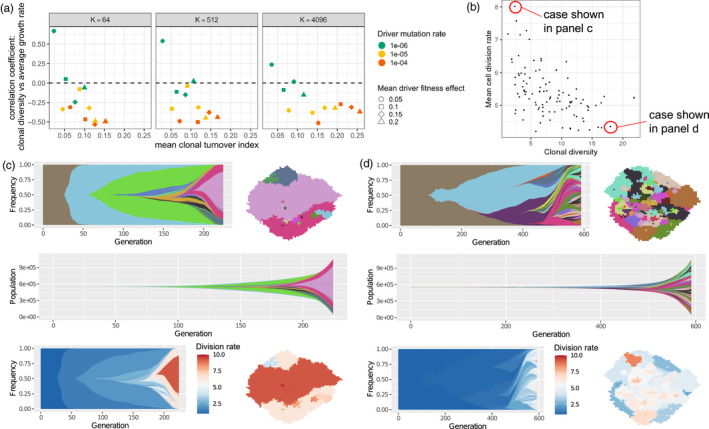
Higher clonal diversity can be associated with slower future tumour growth. (a) Correlation coefficients between clonal diversity (measured at a tumour size of 250,000 cells) and subsequent tumour growth rate, plotted against mean clonal turnover index. Each point represents a cohort of 100 simulated tumours. (b) An example of a tumour cohort exhibiting negative correlation between clonal diversity (measured at a tumour size of 750,000 cells) and mean cell division rate (Spearman's correlation coefficient − 0.55; *p* < 10^−8^). (c) A simulated tumour from this cohort exhibiting a succession of selective sweeps, resulting in high mean cell division rate, high growth rate and low clonal diversity. Top left is a Muller plot in which colours represent clones with distinct combinations of driver mutations (the original clone is grey‐brown; subsequent clones are coloured using a recycled palette of 26 colours). Descendant clones are shown emerging from inside their parents. Top right is a spatial plot of the tumour at the endpoint time, in which each pixel corresponds to a deme containing approximately *K* cells, coloured according to the most abundant clone within the deme. The middle row shows a Muller plot of clone sizes, rather than frequencies. In the bottom row, clones in the Muller and spatial plots are coloured by cell division rate. (d) A simulated tumour from the same cohort exhibiting low mean cell division rate, low growth rate and high clonal diversity due to extensive clonal interference. Parameter values in panels b–d are *K* = 512, *μ* = 10^−5^, *s* = 0.2. Muller plots were drawn using the ggmuller R package (Noble, 2019b)

A potential explanation for negative diversity‐growth correlation, typically observed when clonal diversity is measured at larger tumour sizes (Figure 1b), is the occurrence of selective sweeps that purge heterogeneity. To test this hypothesis, we use a clonal turnover index (see Section 2) to quantify changes in clone frequencies over time. As expected, clonal diversity, when measured at an intermediate tumour size, is positively correlated with future tumour growth rate only in cohorts that have low clonal turnover, whereas correlations are negative in cohorts that have high clonal turnover (Figure 2a). Low clonal turnover corresponds to one or more of three factors: low driver mutation rate, low driver fitness effect, and/or small deme carrying capacity (Figure S2).

In cohorts with high clonal turnover, we observe very similar negative correlations between clonal diversity and contemporaneous mean cell division rate (Figures 2b and S3). Examining the evolutionary dynamics of individual tumours within such cohorts, we find that the combination of low clonal diversity, high mean cell division rate and high tumour growth rate is characteristic of a succession of selective sweeps (Figure 2c). Conversely, high clonal diversity, low mean cell division rate and low tumour growth rate result from clonal interference between less well‐adapted clones (Figure 2d).

### 3.4 Clonal diversity is positively correlated with future growth rate among tumours with diverse driver mutation rates

It is reasonable to expect that, in reality, even tumours of the same size and type (morphology and anatomical location) vary in their underlying biological parameters because of intrinsic and microenvironmental factors. In particular, genomic instability and mutation burden are highly variable within cancer types (Chalmers et al., 2017) and—at least across cancer types—the number of driver mutations per tumour generally increases with ﻿total mutation burden (Martincorena et al., 2017). To examine the consequences of such variation, we next analyse cohorts containing tumours with differing values of the driver mutation rate, μ.

**Figure 3:**
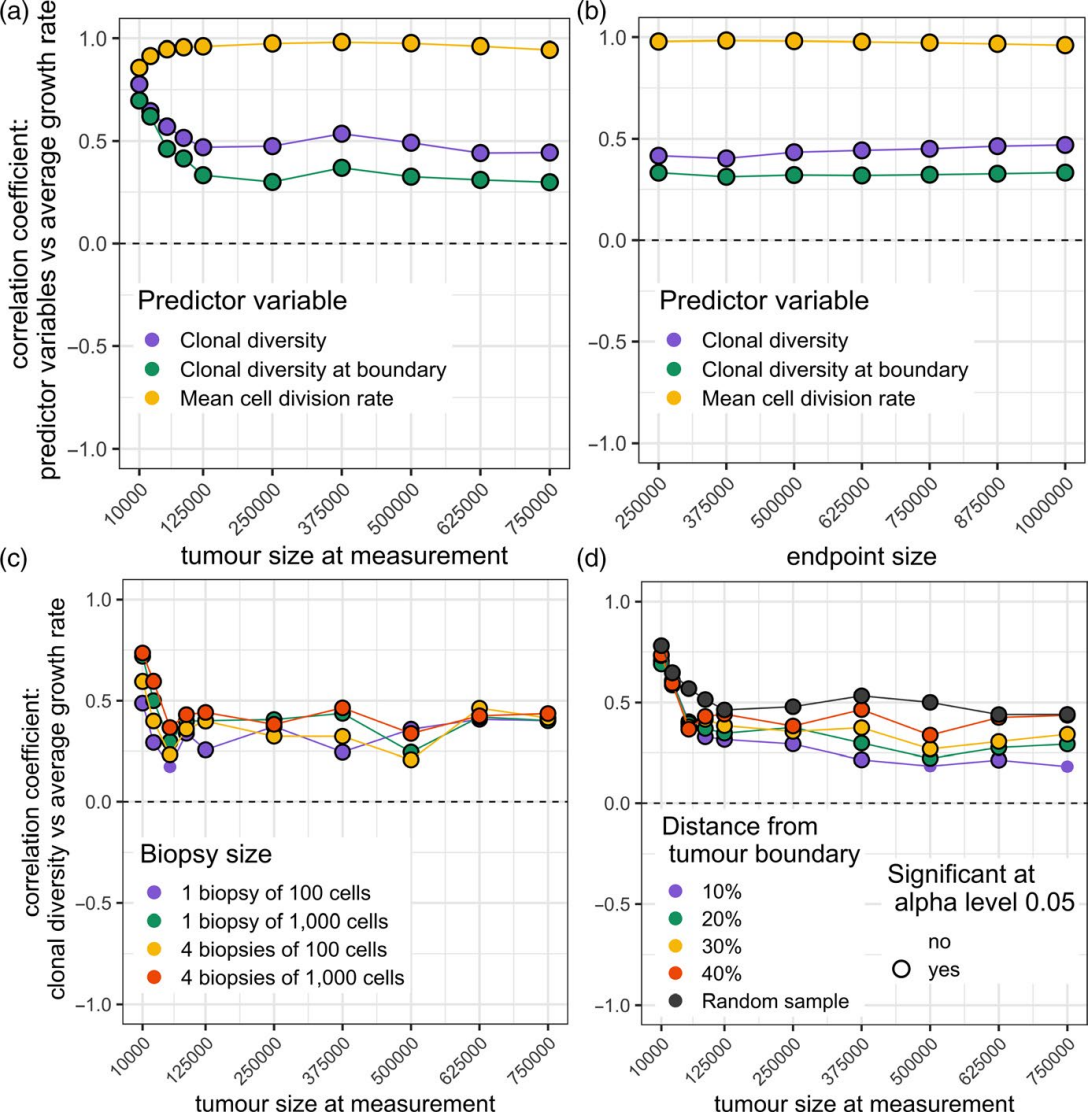
Correlation coefficients for cohorts of tumours with diverse driver mutation rates. (a) Correlation coefficients between predictor variables and subsequent tumour growth rate, for different measurement sizes. (b) Correlation coefficients between predictor variables (measured at a tumour size of 125,000 cells) and subsequent tumour growth rate, for different endpoint sizes. (c) Correlation coefficients between future tumour growth rate and clonal diversity, measured in biopsy samples of different number and size (at a depth of 40% from the tumour edge), for different measurement sizes. (d) Correlation coefficients between future tumour growth rate and clonal diversity, measured in biopsy samples taken at different depths relative to the tumour edge (four biopsy samples, each of 1,000 cells, are taken from each tumour), for different measurement sizes. Correlation coefficients that significantly differ from zero (*p* < .05) are indicated by black rings. In all plots, each point represents a cohort of 100 simulated tumours; lines are added only to guide the eye. Parameter values are *K* = 512 and *s* = 0.1. The driver mutation rate of each tumour is chosen by sampling a random value *X* from a continuous uniform distribution between 4 and 6 and then setting *μ* = 10^−^ *^X^*

In such cohorts, we find that future growth rate is consistently positively correlated with clonal diversity (Figure 3a, blue curves; Figure S4a). As previously discussed, cells near the tumour edge have a disproportionate influence on tumour growth rate, whereas observing the entire tumour provides more reliable information about the mutation rate. When mutation rate substantially differs between tumours, information about this rate has high predictive value. Accordingly, we find that in this case correlations with future growth rate are stronger when diversity is measured across the entire tumour, rather than only at the tumour edge (Figure 3a, blue vs. green curves). In either case, correlation coefficients are insensitive to varying the endpoint size (Figure 3b). These results demonstrate that parameter variability within a cohort can transform diversity‐growth correlations, provided that intrinsic, deterministic differences between tumours outweigh the stochastic differences that emerge during evolution. Nevertheless, we find that, as a predictor of future growth rate, clonal diversity is always substantially inferior to mean cell division rate (Figure 3a, yellow curves; Figure S4a).

When tumours differ in the mean driver fitness effect (parameter *s*) but not in driver mutation rate (*μ*), diversity‐growth correlations instead follow the same pattern as when parameter values do not vary (Figure S5).

### 3.5 The predictive value of clonal diversity is robust to biopsy sampling error

Clinical evaluations of intratumour heterogeneity are typically based on examining cells in one or more relatively small biopsy samples. To assess how sampling error affects our results, we repeated our analyses using clonal diversity measurements derived only from biopsies (see Section 2). When driver mutation rate varies within cohorts, results based on biopsies are similar to those based on all tumour cells, except when the biopsy size is much below 1,000 cells (Figures 3c and S4b). Also when driver mutation rate varies within cohorts, diversity‐growth correlations are stronger when biopsy samples are taken from inside the tumour, rather than from the edge (Figures 3d and S4c).

### 3.6 Clonal diversity as a predictor of progression‐free survival

**Figure 4:**
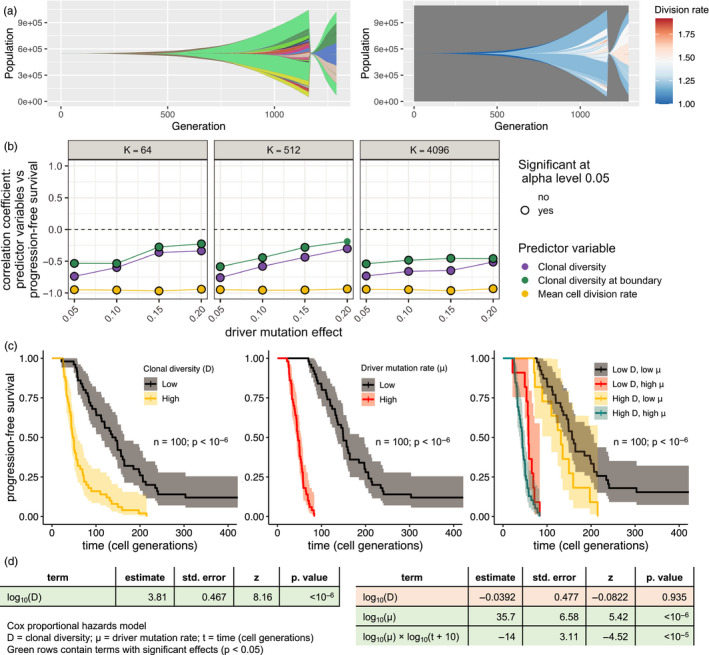
Forecasting progression‐free survival in tumours with diverse driver mutation rates. (a) Muller plots for a simulated tumour that is treated and regrows to its pretreatment size. In the left panel, colours represent clones with distinct combinations of driver mutations (using a recycled palette of 26 colours). Descendant clones are shown emerging from inside their parents. In the right panel, clones are coloured by cell division rate. (b) Correlation coefficients between predictor variables measured immediately before treatment and progression‐free survival (time to regrow to pretreatment size). (c) Kaplan–Meier curves for progression‐free survival in a cohort of tumours (parameter values *K* = 512 and *s* = 0.05). Tumours are grouped by pretreatment clonal diversity (first panel); driver mutation rate (second panel); or both pretreatment clonal diversity and driver mutation rate (third panel). For grouping, values above and below the median are classed as high and low, respectively. The endpoint size is one million cells. The driver mutation rate of each tumour is chosen by sampling a random value *X* from a continuous uniform distribution between 4 and 6 and then setting *μ* = 10^−^ *^X^*. To facilitate comparison, colours are consistent with figure 7 of Turajlic et al. (2018). (d) Results of two alternative survival analyses using the Cox proportional hazards model (see Section 2 for further details)

To investigate forecasting response to treatment, we extend our computational model to include the acquisition of resistance mutations at a rate proportional to the driver mutation rate. We simulate treatment application at the endpoint tumour size by removing all tumour cells that lack resistance mutations and we measure progression‐free survival as the time taken to regrow to the endpoint size (Figure 4a). If all cells are sensitive to treatment then the tumour is eliminated and progression‐free survival is assumed to outlast the observation period.

In cohorts of tumours with differing driver mutation rates, we find that clonal diversity measured immediately before treatment is negatively correlated with progression‐free survival (Figure 4b). This is an expected corollary of our previous results because the probability of a cell being resistant is independent of its division rate, and hence the mean cell division rate immediately before treatment equates to its expected value immediately after treatment. In other words, tumours that were growing relatively fast before treatment are also likely to rebound quickly after treatment.

The prognostic value of clonal diversity can be more thoroughly understood using survival analysis. Mimicking the approach taken in the TRACERx Renal study of ﻿clear‐cell renal cell carcinoma (Turajlic et al., 2018), we first divide a cohort of 100 tumours into subsets with high or low pretreatment clonal diversity and high or low driver mutation rate. The hazard rate for progression is then higher in the high‐diversity subset, both among all tumours (log‐rank test *p* < 10^–6^) and among those with low driver mutation rates (*p* = .031; Figure 4c).

When we instead treat pretreatment clonal diversity as a continuous explanatory variable, we likewise find a significant effect on progression‐free survival (Cox proportional hazards model *p* < 10^–6^). However, if driver mutation rate is also included as a continuous variable then the effect of clonal diversity disappears (*p* = .94; Figure 4d). Hence different results are obtained depending on whether the explanatory variables are treated as discrete or continuous. This apparent discrepancy can be explained as an effect of covariance: within the low‐mutation rate subset, tumours with low clonal diversity also tend to be those with especially low‐mutation rates.

Similarly, if we combine cohorts with different mean driver mutation fitness effects (in the manner of a pan‐cancer analysis) then we find that, although both pretreatment clonal diversity and driver mutation rate predict progression‐free survival (*n* = 400; *p* < 10^–6^), the effect of clonal diversity disappears when mean cell division rate is included in the statistical model (*p* = .78; Figure S6). In summary, these results indicate that clonal diversity is a useful predictor of progression‐free survival only inasmuch as it is a proxy for importantly varying biological parameters (such as driver mutation rate and mean cell division rate) that cannot themselves be precisely measured or inferred.

## 4 DISCUSSION

Intratumour heterogeneity is considered a promising prognostic biomarker because it relates to the evolutionary processes that drive tumour progression (Alizadeh et al., 2015; Jamal‐Hanjani et al., 2015; Maley et al., 2017; Marusyk et al., 2012; Polyak, 2014). The general nature of this relationship has, however, proven difficult to characterize. By simulating tumour evolution under different conditions in numerous virtual patient cohorts, here we have taken first steps towards disentangling when, why and how clonal diversity (a particular form of intratumour heterogeneity) predicts tumour growth rate and clinical progression.

We have identified three mutually reinforcing factors that can lead to positive correlations between clonal diversity and future tumour growth. First, positive diversity‐growth correlations are seen when clonal diversity is measured at an early stage of tumour progression. This is because high diversity in a small tumour indicates the presence of mutations that are under selection, and that will eventually accelerate tumour growth, but that have not yet had time to fix. Second, clonal diversity, even when measured at a later stage, can positively correlate with future growth rate provided that tumour spatial structure and/or evolutionary parameters sufficiently restrict the rate of clonal turnover, which otherwise purges diversity within more aggressive tumours. Finally, positive diversity‐growth correlations can arise in cohorts of tumours that have differing rates of acquiring beneficial (driver) mutations—for example due to variation in genomic instability—because higher diversity then correlates with higher driver mutation rate, which in turn predicts faster tumour growth. In the absence of all three of these factors, we find that the correlation between clonal diversity and future tumour growth rate is typically negative. These results are robust to biopsy sampling error and readily extend to forecasting progression‐free survival after treatment.

Although the influence of stochastic factors fundamentally limits predictability in our model—as is the case in real cancers (Lipinski et al., 2016)—we find that tumour growth forecasts can be remarkably reliable. Due to complex processes at multiple spatial and temporal scales, forecasts of weather and of many kinds of ecological dynamics become progressively less accurate as they reach further into the future (Petchey et al., 2015). Tumour growth in our model conforms to a very different pattern, such that forecast accuracy is unchanged or even improves as the projection period lengthens (Figures 3b and S4b). This is because, in a growing population, early mutations are likely to reach higher frequency and hence be more influential than later mutations (which arise in a larger population, typically further from the tumour edge, and encounter more clonal interference), yet even early mutations have somewhat delayed effects on tumour growth, due to the time required for clonal expansion. A tumour's long‐term growth trajectory is mostly determined at an early stage, even as driver mutations occur increasingly often over time.

In their own study cohort and in the larger TCGA kidney cancer cohort, the TRACERx Renal consortium found that low intratumour heterogeneity correlates with longer progression‐free and overall survival times when tumours have low genomic instability, but not when tumours have high genomic instability (Turajlic et al., 2018). Our computational model generates a similar pattern (Figures 4c and S7) and thus provides an explanation for clinical observations. From a clinical perspective, however, the crucial question is whether new prognostic biomarkers can outperform the status quo. The TRACERx Renal consortium found that the predictive power of intratumour heterogeneity and genomic instability remained significant after adjusting for known prognostic variables (including stage and grade) in the TCGA kidney cancer cohort but not in their own smaller cohort of 100 tumours (Turajlic et al., 2018). Consistently, in our model results, forecasting growth rate and progression‐free survival from clonal diversity is inferior to forecasting based on mean cell division rate (a correlate of cancer grade) and the predictive power of clonal diversity vanishes if additional explanatory variables can be accounted for. Notwithstanding this theoretical limitation, a key part of the appeal of clonal diversity as a prognostic biomarker is that, in practice, it is easier to measure precisely than the alternative indices with which it covaries.

Our work contributes to a growing body of work on predicting evolution in complex systems, which is gaining increasing attention because of its potential to provide mechanistic explanations for patterns observed in clinical data and to guide therapeutic interventions (reviewed in Lässig, Mustonen, & Walczak, 2017). For example, using a nonspatial computational model and analysis of high‐throughput sequencing data, Williams et al. (2018) recently inferred the strength of selection in various cancer types and used the results to forecast changes in clonal architecture. Hosseini, Diaz‐Uriarte, Markowetz, and Beerenwinkel (2019) and Diaz‐Uriarte and Vasallo (2019) have instead examined the order of accumulation of driver mutations and found that certain types of fitness landscape lead to more or less predictable tumour evolutionary trajectories. Our approach is both distinct and complementary, in that we seek to describe the stochastic processes of clonal initiation, expansion and interaction in a realistic spatial context, and we focus on generic, clinically relevant predictor variables and outcomes.

We have kept our model simple so as to yield the most general insights. Our work thus has several limitations that motivate further investigation. Even allowing for the fact that we simulate a two‐dimensional slice of a larger three‐dimensional tumour, the endpoint size of one million cells is unrealistically small. This discrepancy might be relatively inconsequential, however, given our finding that forecast accuracy is robust to increasing endpoint size. The essential principles that underlie our results apply in three dimensions just as in two dimensions, but outcomes might differ quantitatively due to reduced clonal interference in the former case. In common with previous computational modelling studies (Bozic et al., 2010; Waclaw et al., 2015), we assume an infinite sites model of evolution. We also assume only very weak diminishing‐returns epistasis. We expect that assuming a finite sites model or stronger diminishing‐returns epistasis—such as observed in long‐term E. coli evolution (Wiser et al., 2013)—would tend to make tumour growth even more deterministic and more predictable. Relatedly, we neglect deleterious mutations and hence do not impose any fitness cost of high genomic instability, a factor that might help explain slower growth of especially heterogeneous tumours (Andor et al., 2015). We have not attempted to investigate how forecast accuracy might be affected by frequency‐dependent cell–cell interactions, variable deme carrying capacity or other forms of microenvironmental heterogeneity. Nor have we tested here whether clonal diversity can predict metastatic potential, which is a crucial factor in overall survival.

An especially promising direction for future research is in combining clonal diversity measurements with information about evolutionary processes, such as the pervasiveness of selective sweeps in a given tumour type across individuals or in a particular tumour within an individual (Maley et al., 2017). Although it is generally infeasible to infer a complete history of clonal turnover, genomic data from even a single time point can potentially be used to infer the strength of selection, time since the most recent common ancestor, and rate of demographic expansion (Alves, Prado‐López, Cameselle‐Teijeiro, & Posada, 2019; Williams et al., 2018). Future work should employ multi‐variable statistical models that incorporate such information about tumour evolution, ecology and demography (Maley et al., 2017). These methods are likely to outperform the simpler models we have examined here. Future studies should also evaluate system‐specific modifications of our model and our biopsy sampling protocol, using multi‐omics data, histopathology image analysis, and cancer staging assays to infer sub‐clonal cell proliferation rates and to characterize cell–cell interactions. By laying the foundations for such projects, the current study comprises a step towards the ultimate goal of personalized cancer evolution forecasts, parametrized with patient‐specific data.

## CONFLICT OF INTEREST

None declared.

## AUTHOR CONTRIBUTIONS

MEH and RN conceived the research question and designed the study. RN designed and created the computational modelling framework. JTB, CLS and RN wrote analysis code, ran simulations and analysed output data. RN wrote the manuscript with contributions from CLS and MEH and drawing from the related master's thesis of JTB.

## Supporting information

Figure S1

Figure S2

Figure S3

Figure S4

Figure S5

Figure S6

Figure S7

Figure S8

## Data Availability

Code used for data analysis and plotting, as well as an informative sample of model output data, can be found at https://github.com/robjohnnoble/forecasting_paper and https://github.com/robjohnnoble/demonanalysis.

## References

[eva13057-bib-0001] Alizadeh, A. A. , Aranda, V. , Bardelli, A. , Blanpain, C. , Bock, C. , Borowski, C. , … Zucman‐Rossi, J. (2015). Toward understanding and exploiting tumor heterogeneity. Nature Medicine, 21(8), 846–853. 10.1038/nm.3915 PMC4785013 26248267

[eva13057-bib-0002] Alves, J. M. , Prado‐López, S. , Cameselle‐Teijeiro, J. M. , & Posada, D. (2019). Rapid evolution and biogeographic spread in a colorectal cancer. Nature Communications, 10(1), 5139 10.1038/s41467-019-12926-8 PMC6853914 31723138

[eva13057-bib-0003] Andor, N. , Graham, T. A. , Jansen, M. , Xia, L. C. , Aktipis, C. A. , Petritsch, C. , … Maley, C. C. (2015). Pan‐cancer analysis of the extent and consequences of intratumor heterogeneity. Nature Medicine, 22(1), 105–113. 10.1038/nm.3984 PMC4830693 26618723

[eva13057-bib-0004] Bozic, I. , Antal, T. , Ohtsuki, H. , Carter, H. , Kim, D. , Chen, S. , … Nowak, M. A. (2010). Accumulation of driver and passenger mutations during tumor progression. Proceedings of the National Academy of Sciences of the United States of America, 107, 18545–18550. 10.1073/pnas.1010978107 20876136 PMC2972991

[eva13057-bib-0005] Bozic, I. , Paterson, C. , & Waclaw, B. (2019). On measuring selection in cancer from subclonal mutation frequencies. PLOS Computational Biology, 15(9), e1007368 10.1371/journal.pcbi.1007368 31557163 PMC6788714

[eva13057-bib-0006] Burrell, R. A. , McGranahan, N. , Bartek, J. , & Swanton, C. (2013). The causes and consequences of genetic heterogeneity in cancer evolution. Nature, 501, 338–345. 10.1038/nature12625 24048066

[eva13057-bib-0007] Chalmers, Z. R. , Connelly, C. F. , Fabrizio, D. , Gay, L. , Ali, S. M. , Ennis, R. , … Frampton, G. M. (2017). Analysis of 100,000 human cancer genomes reveals the landscape of tumor mutational burden. Genome Medicine, 9(1), 1–14. 10.1186/s13073-017-0424-2 28420421 PMC5395719

[eva13057-bib-0008] Cross, W. , Graham, T. A. , & Wright, N. A. (2016). New paradigms in clonal evolution: Punctuated equilibrium in cancer. The Journal of Pathology, 240(2), 126–136. 10.1002/path.4757 27282810

[eva13057-bib-0009] Dhawan, A. , Graham, T. , & Fletcher, A. G. (2016). A computational modelling approach for deriving biomarkers to predict cancer risk in premalignant disease. Cancer Prevention Research, 24(4), 1–42. 10.1158/1940-6207.CAPR-15-0248 26851234

[eva13057-bib-0010] Diaz‐Uriarte, R. , & Vasallo, C. (2019). Every which way? On predicting tumor evolution using cancer progression models. PLOS Computational Biology, 15(8), e1007246 10.1371/journal.pcbi.1007246 31374072 PMC6693785

[eva13057-bib-0011] Gerlinger, M. , Rowan, A. J. , Horswell, S. , Larkin, J. , Endesfelder, D. , Gronroos, E. , … Swanton, C. (2012). Intratumor heterogeneity and branched evolution revealed by multiregion sequencing. New England Journal of Medicine, 366, 1467–1476. 10.1056/NEJMoa1113205 22397650 PMC4878653

[eva13057-bib-0012] Gillespie, D. T. (1977). Exact stochastic simulation of coupled chemical reactions. The Journal of Physical Chemistry, 81, 2340–2361. 10.1021/j100540a008

[eva13057-bib-0013] Greaves, M. , & Maley, C. C. (2012). Clonal evolution in cancer. Nature, 481, 306–313. 10.1038/nature10762 22258609 PMC3367003

[eva13057-bib-0014] Hanahan, D. , & Weinberg, R. A. (2011). Hallmarks of cancer: The next generation. Cell, 144, 646–674. 10.1016/j.cell.2011.02.013 21376230

[eva13057-bib-0015] Hosseini, S.‐R. , Diaz‐Uriarte, R. , Markowetz, F. , & Beerenwinkel, N. (2019). Estimating the predictability of cancer evolution. Bioinformatics, 35(14), i389–i397. 10.1093/bioinformatics/btz332 31510665 PMC6612861

[eva13057-bib-0016] Jamal‐Hanjani, M. , Quezada, S. A. , Larkin, J. , & Swanton, C. (2015). Translational implications of tumor heterogeneity. Clinical Cancer Research, 21(12), 1258–1266. 10.1158/1078-0432.CCR-14-1429 25770293 PMC4374162

[eva13057-bib-0017] Jamal‐Hanjani, M. , Wilson, G. A. , McGranahan, N. , Birkbak, N. J. , Watkins, T. B. K. , Veeriah, S. , … Swanton, C. (2017). Tracking the evolution of non–small‐cell Lung cancer. New England Journal of Medicine, 376(22), 2109–2121. 10.1056/NEJMoa1616288 28445112

[eva13057-bib-0018] Kassambara, A. , Kosinski, M. , & Biecek, P. (2019). survminer: Drawing Survival Curves using 'ggplot2'. R package version 0.4.6. Retrieved from https://CRAN.R‐project.org/package=survminer

[eva13057-bib-0019] Lang, G. I. , Rice, D. P. , Hickman, M. J. , Sodergren, E. , Weinstock, G. M. , Botstein, D. , & Desai, M. M. (2013). Pervasive genetic hitchhiking and clonal interference in forty evolving yeast populations. Nature, 500, 571–574. 10.1038/nature12344 23873039 PMC3758440

[eva13057-bib-0020] Lässig, M. , Mustonen, V. , & Walczak, A. M. (2017). Predicting evolution. Nature Ecology & Evolution, 1(3), 0077. 10.1038/s41559-017-0077 28812721

[eva13057-bib-0021] Lipinski, K. A. , Barber, L. J. , Davies, M. N. , Ashenden, M. , Sottoriva, A. , & Gerlinger, M. (2016). Cancer evolution and the limits of predictability in precision cancer medicine. Trends in Cancer, 2, 49–63. 10.1016/j.trecan.2015.11.003 26949746 PMC4756277

[eva13057-bib-0022] Maley, C. C. , Aktipis, A. , Graham, T. A. , Sottoriva, A. , Boddy, A. M. , Janiszewska, M. , … Shibata, D. (2017). Classifying the evolutionary and ecological features of neoplasms. Nature Reviews Cancer, 17(10), 605–619. 10.1038/nrc.2017.69 28912577 PMC5811185

[eva13057-bib-0023] Maley, C. C. , Galipeau, P. C. , Finley, J. C. , Wongsurawat, V. J. , Li, X. , Sanchez, C. A. , Paulson, T. G. , … Reid, B. J. (2006). Genetic clonal diversity predicts progression to esophageal adenocarcinoma. Nature Genetics, 38, 468–473. 10.1038/ng1768 16565718

[eva13057-bib-0024] Martens, E. A. , Kostadinov, R. , Maley, C. C. , & Hallatschek, O. (2011). Spatial structure increases the survival time for cancer. New Journal of Physics, 13, 115014 10.1088/1367-2630/13/11/115014 22707911 PMC3375912

[eva13057-bib-0025] Martincorena, I. , Raine, K. M. , Gerstung, M. , Dawson, K. J. , Haase, K. , Van Loo, P. , … Campbell, P. J. (2017). Universal patterns of selection in cancer and somatic tissues. Cell, 171(5), 1029–1041.e21. 10.1016/j.cell.2017.09.042 29056346 PMC5720395

[eva13057-bib-0026] Martinez, P. , Timmer, M. R. , Lau, C. T. , Calpe, S. , Sancho‐Serra, M. D. C. , Straub, D. , … Krishnadath, K. K. (2016). Dynamic clonal equilibrium and predetermined cancer risk in Barrett's oesophagus. Nature Communications, 7, 12158 10.1038/ncomms12158 PMC4992167 27538785

[eva13057-bib-0027] Marusyk, A. , Almendro, V. , & Polyak, K. (2012). Intra‐tumour heterogeneity: A looking glass for cancer? Nature Reviews Cancer, 12, 323–334. 10.1038/nrc3261 22513401

[eva13057-bib-0028] Merlo, L. M. F. , Shah, N. A. , Li, X. , Blount, P. L. , Vaughan, T. L. , Reid, B. J. , & Maley, C. C. (2010). A comprehensive survey of clonal diversity measures in Barrett's esophagus as biomarkers of progression to esophageal adenocarcinoma. Cancer Prevention Research, 3, 1388–1397. 10.1158/1940-6207.CAPR-10-0108 20947487 PMC3004782

[eva13057-bib-0029] Michor, F. , Frank, S. A. , May, R. M. , Iwasa, Y. , & Nowak, M. A. (2003). Somatic selection for and against cancer. Journal of Theoretical Biology, 225, 377–382. 10.1016/S0022-5193(03)00267-4 14604590

[eva13057-bib-0030] Noble, R. (2019a). demon: Deme‐based oncology model. Retrieved from https://github.com/robjohnnoble/demon_model.

[eva13057-bib-0031] Noble, R. (2019b). ggmuller: Create muller plots of evolutionary dynamics. Retrieved from https://cran.r‐project.org/package=ggmuller.

[eva13057-bib-0032] Noble, R. , Burri, D. , Kather, J. N. , & Beerenwinkel, N. (2019) Spatial structure governs the mode of tumour evolution. bioRxiv. 10.1101/586735. PMC8825284 34949822

[eva13057-bib-0033] Park, S. Y. , Gönen, M. , Kim, H. J. , Michor, F. , & Polyak, K. (2010). Cellular and genetic diversity in the progression of in situ human breast carcinomas to an invasive phenotype. Journal of Clinical Investigation, 120(2), 636–644. 10.1172/JCI40724 20101094 PMC2810089

[eva13057-bib-0034] Petchey, O. L. , Pontarp, M. , Massie, T. M. , Kéfi, S. , Ozgul, A. , Weilenmann, M. , … Pearse, I. S. (2015). The ecological forecast horizon, and examples of its uses and determinants. Ecology Letters, 18(7), 597–611. 10.1111/ele.12443 25960188 PMC4676300

[eva13057-bib-0035] Polyak, K. (2014). Tumor heterogeneity confounds and illuminates: A case for Darwinian tumor evolution. Nature Medicine, 20, 344–346. 10.1038/nm.3518 24710378

[eva13057-bib-0036] Robertson‐Tessi, M. , & Anderson, A. R. A. (2015). Big Bang and context‐driven collapse. Nature Genetics, 47, 196–197. 10.1038/ng.3231 25711865

[eva13057-bib-0037] Rye, I. H. , Trinh, A. , Sætersdal, A. B. , Nebdal, D. , Lingjærde, O. C. , Almendro, V. , … Russnes, H. G. (2018). Intratumor heterogeneity defines treatment‐resistant HER2+ breast tumors. Molecular Oncology, 12(11), 1838–1855. 10.1002/1878-0261.12375 30133130 PMC6210052

[eva13057-bib-0038] Schwarz, R. F. , Ng, C. K. Y. , Cooke, S. L. , Newman, S. , Temple, J. , Piskorz, A. M. , … Brenton, J. D. (2015). Spatial and temporal heterogeneity in high‐grade serous ovarian cancer: A phylogenetic analysis. PLoS Medicine, 12, e1001789 10.1371/journal.pmed.1001789 25710373 PMC4339382

[eva13057-bib-0039] Sun, R. , Hu, Z. , Sottoriva, A. , Graham, T. A. , Harpak, A. , Ma, Z. , … Curtis, C. (2017). Between‐region genetic divergence reflects the mode and tempo of tumor evolution. Nature Genetics, 49(7), 1015–1024. 10.1038/ng.3891 28581503 PMC5643198

[eva13057-bib-0040] Therneau, T. (2015). A package for survival analysis in S. version 2.38. Retrieved from https://CRAN.R‐project.org/package=survival.

[eva13057-bib-0041] Turajlic, S. , Sottoriva, A. , Graham, T. , & Swanton, C. (2019). Resolving genetic heterogeneity in cancer. Nature Reviews Genetics, 20(7), 404–416. 10.1038/s41576-019-0114-6 30918367

[eva13057-bib-0042] Turajlic, S. , Xu, H. , Litchfield, K. , Rowan, A. , Horswell, S. , Chambers, T. , … Swanton, C. (2018). Deterministic evolutionary trajectories influence primary tumor growth: TRACERx renal. Cell, 173(3), 595–610.e11. 10.1016/j.cell.2018.03.043 29656894 PMC5938372

[eva13057-bib-0043] Venkatesan, S. , & Swanton, C. (2016). Tumor evolutionary principles: How intratumor heterogeneity influences cancer treatment and outcome. ASCO Educational Book, 36, 141–149. 10.1200/EDBK_158930 27249716

[eva13057-bib-0044] Waclaw, B. , Bozic, I. , Pittman, M. E. , Hruban, R. H. , Vogelstein, B. , & Nowak, M. A. (2015). Spatial model predicts dispersal and cell turnover cause reduced intra‐tumor heterogeneity. Nature, 525, 261–267. 10.1038/nature14971 26308893 PMC4782800

[eva13057-bib-0045] Williams, M. J. , Werner, B. , Graham, T. A. , & Sottoriva, A. (2016). Functional versus non‐functional intratumor heterogeneity in cancer. Molecular & Cellular Oncology, 3(4), e1162897 10.1080/23723556.2016.1162897 27652316 PMC4972105

[eva13057-bib-0046] Williams, M. J. , Werner, B. , Heide, T. , Curtis, C. , Barnes, C. P. , Sottoriva, A. , & Graham, T. A. (2018). Quantification of subclonal selection in cancer from bulk sequencing data. Nature Genetics, 50(6), 895–903. 10.1038/s41588-018-0128-6 29808029 PMC6475346

[eva13057-bib-0047] Wiser, M. J. , Ribeck, N. , & Lenski, R. E. (2013). Long‐term dynamics of adaptation in asexual populations. Science, 342(6164), 1364–1367. 10.1126/science.1243357 24231808

[eva13057-bib-0048] Yankeelov, T. E. , Quaranta, V. , Evans, K. J. , & Rericha, E. C. (2015). Toward a science of tumor forecasting for clinical oncology. Cancer Research, 75, 918–923. 10.1158/0008-5472.CAN-14-2233 25592148 PMC4359948

