# Supplementary figures and images for: When, why and how tumour clonal diversity predicts survival
Source: Evol Appl. 2020 Jul 18;13(7):1558–68. doi: 10.1111/eva.13057 (PMC7428820; doi:10.1111/eva.13057)

Deme size = 64

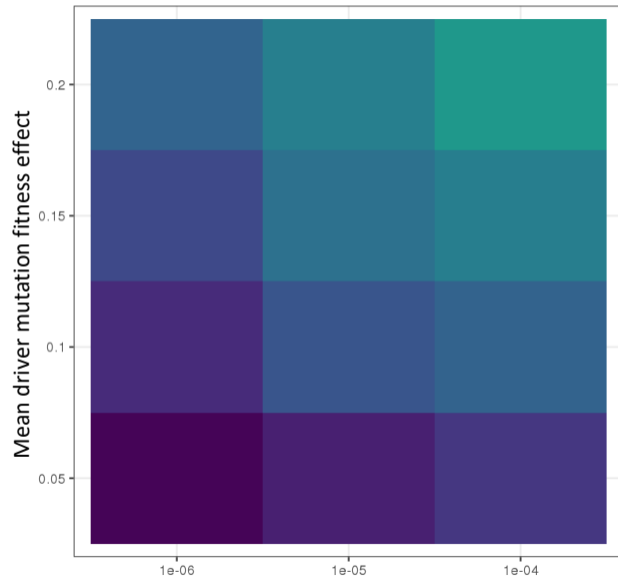

Deme size = 512

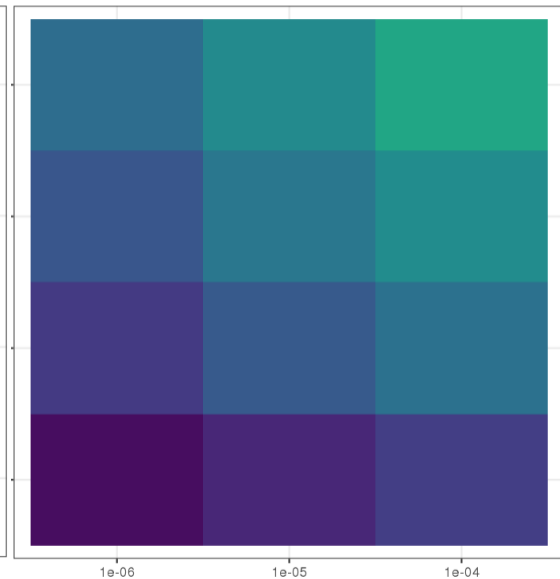

Deme size = 4096

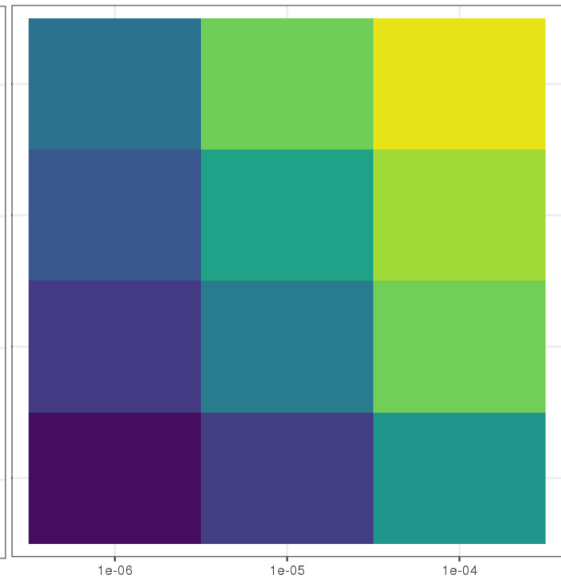

Median clonal turnover

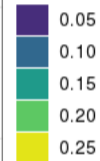

Supplement: Supplementary file 2 — Figure S2 [file EVA-13-1558-s002.pdf]

correlation coefficient:  
clonal diversity vs mean cell division rate

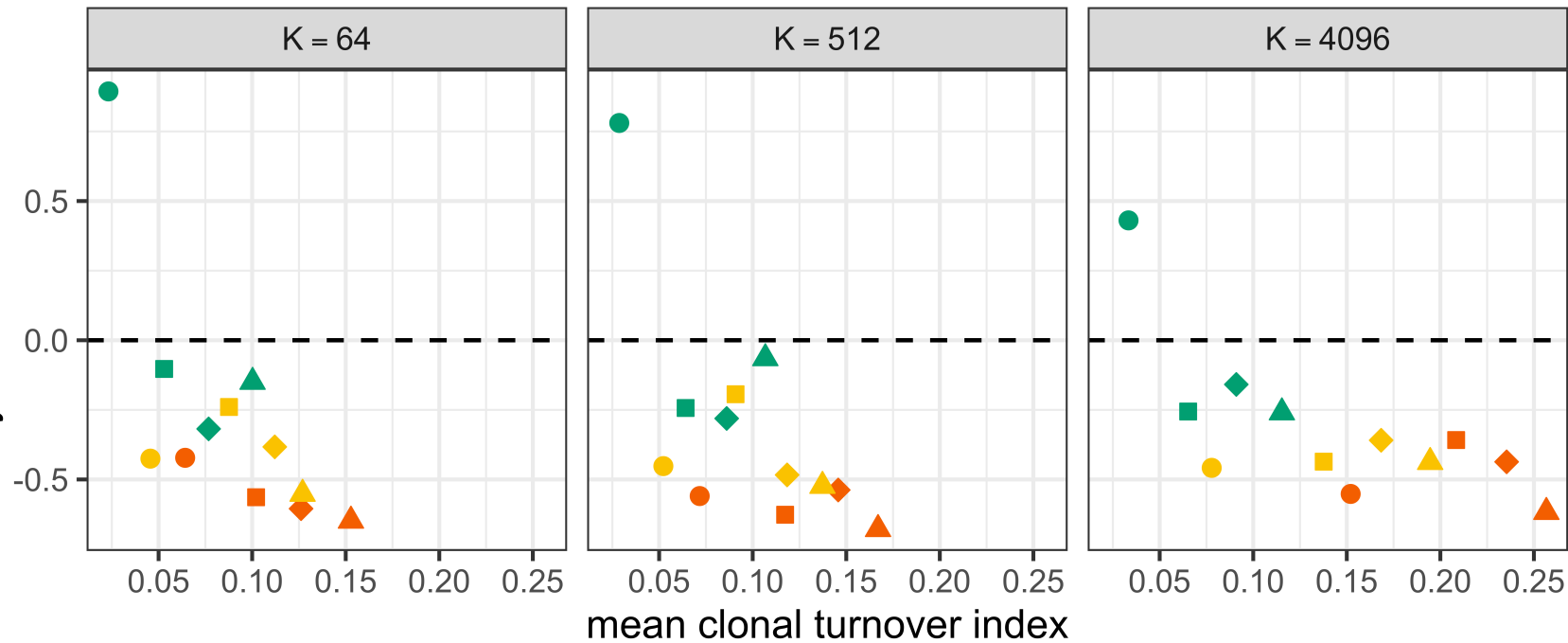

Supplement: Supplementary file 3 — Figure S3 [file EVA-13-1558-s003.pdf]

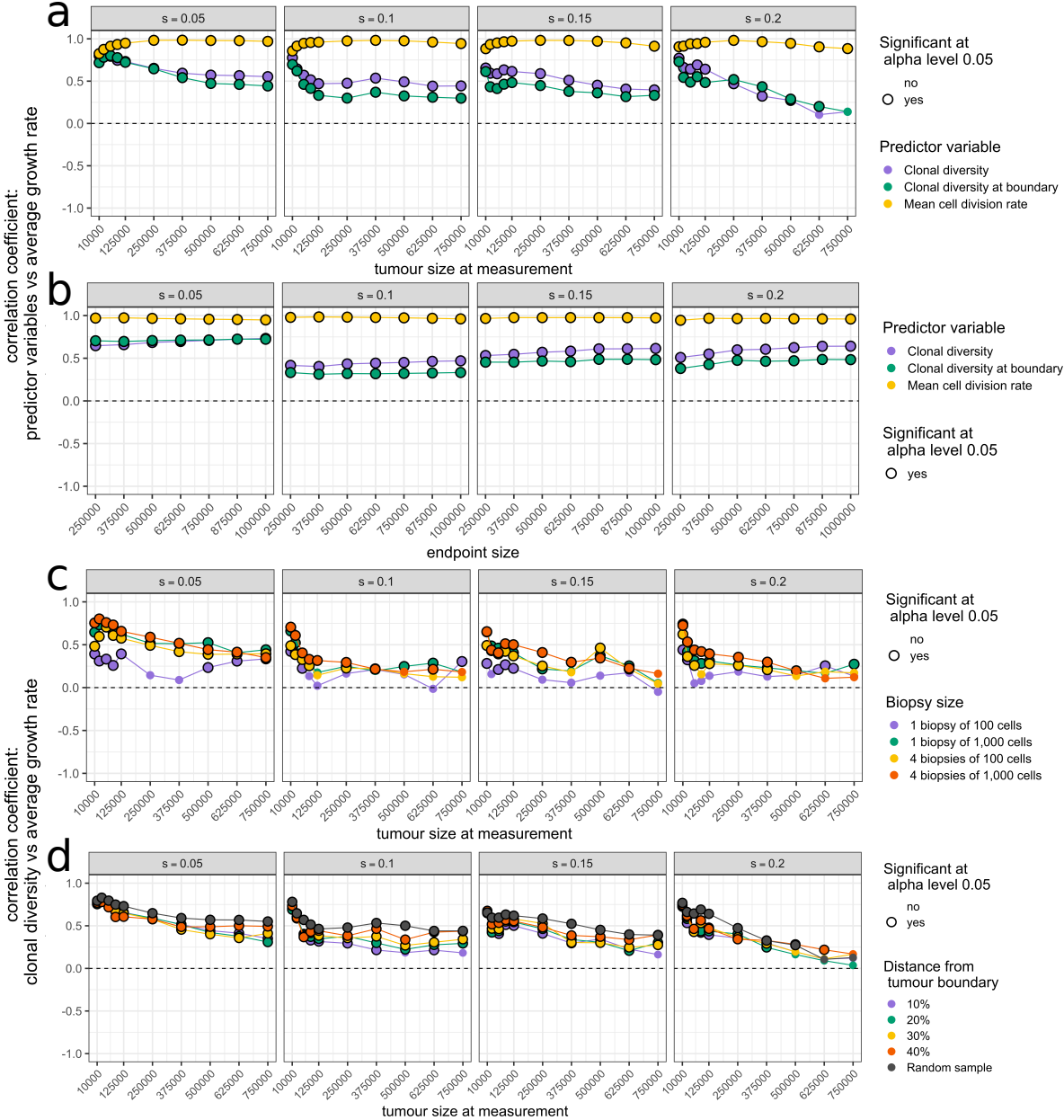

Supplement: Supplementary file 4 — Figure S4 [file EVA-13-1558-s004.pdf]

correlation coefficient:  
predictor variables vs average growth rate

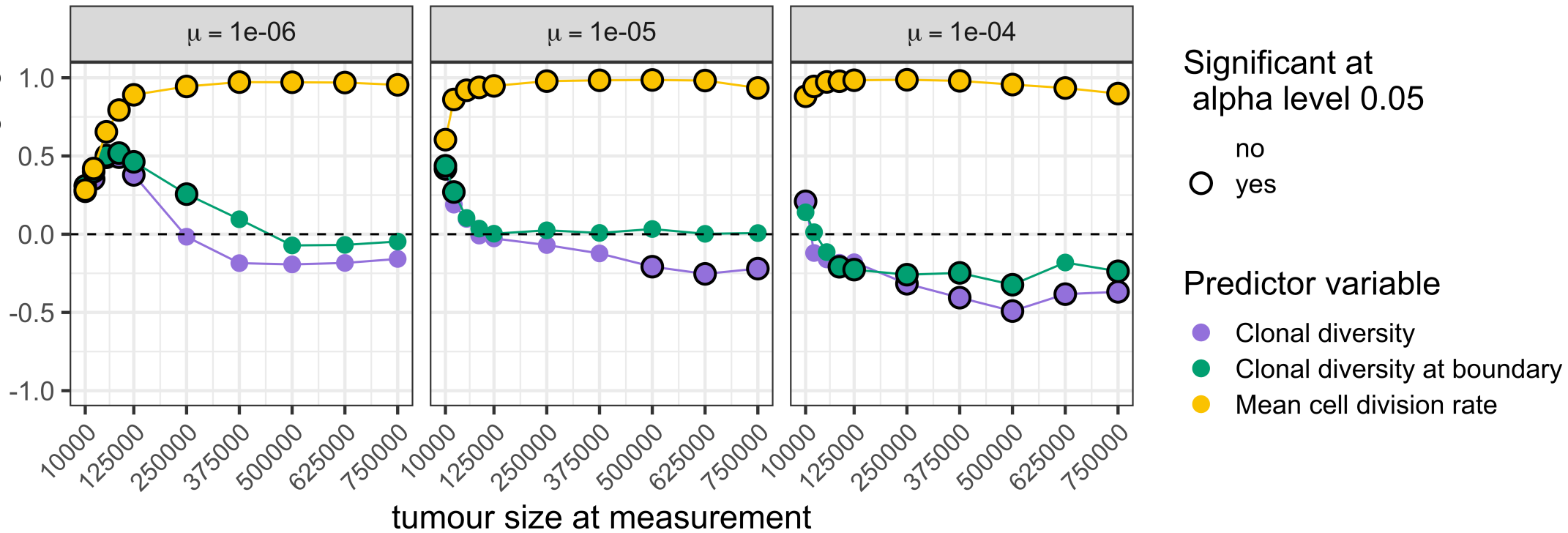

Supplement: Supplementary file 5 — Figure S5 [file EVA-13-1558-s005.pdf]

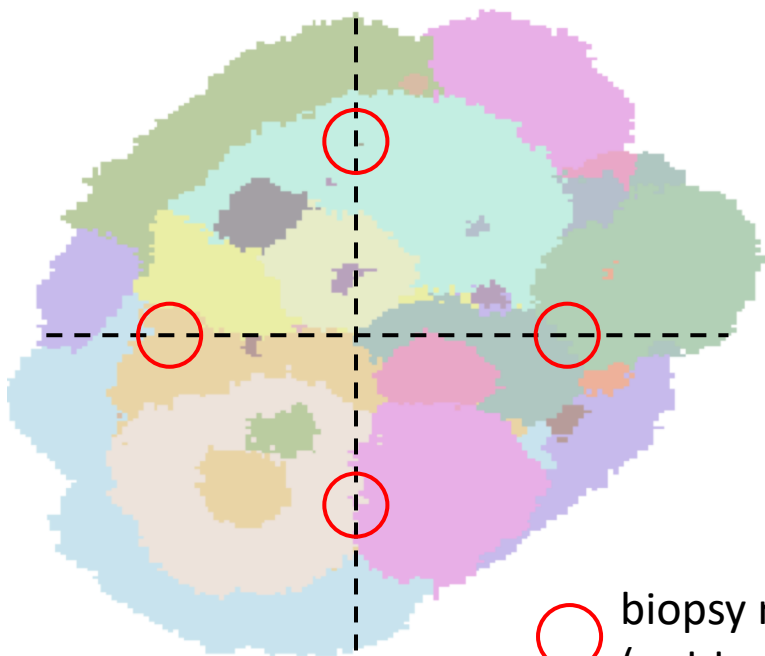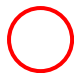

biopsy region  
(not to scale)

Supplement: Supplementary file 6 — Figure S6 [file EVA-13-1558-s006.pdf]

correlation coefficient:  
predictor variables vs  
progression-free survival

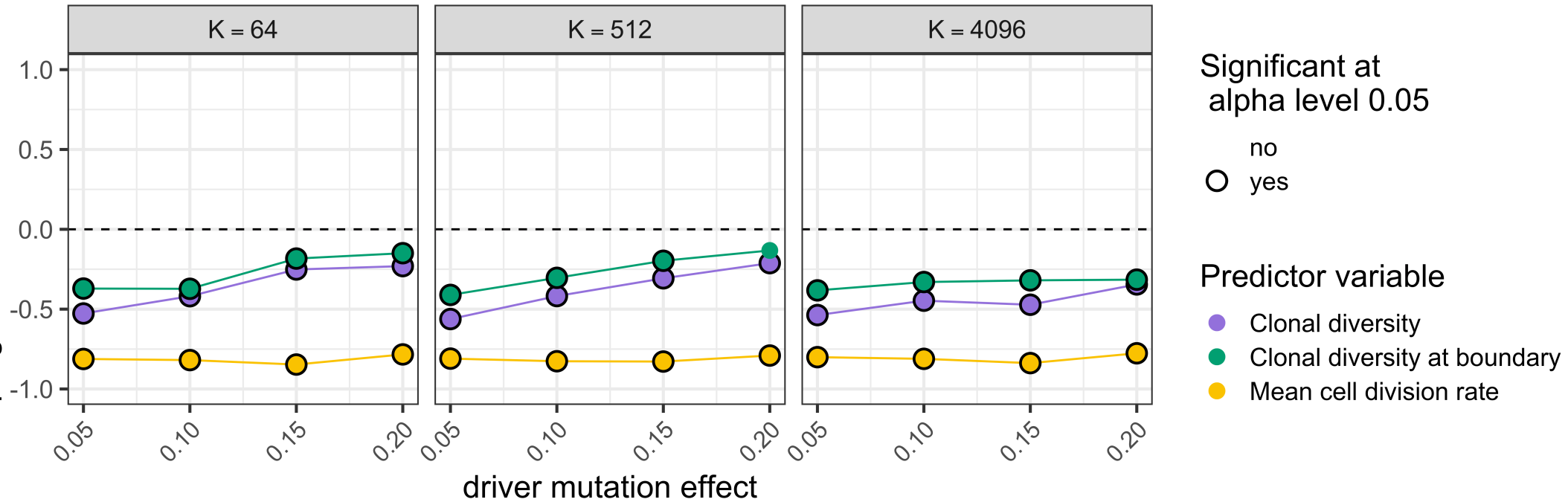

Supplement: Supplementary file 8 — Figure S8 [file EVA-13-1558-s008.pdf]
