# Supplementary material for: When, why and how tumour clonal diversity predicts survival
Source: Evol Appl. 2020 Jul 18;13(7):1558–68. doi: 10.1111/eva.13057 (PMC7428820; doi:10.1111/eva.13057)
Supplement: Supplementary file 1 — Figure S1 [file EVA-13-1558-s001.pdf]

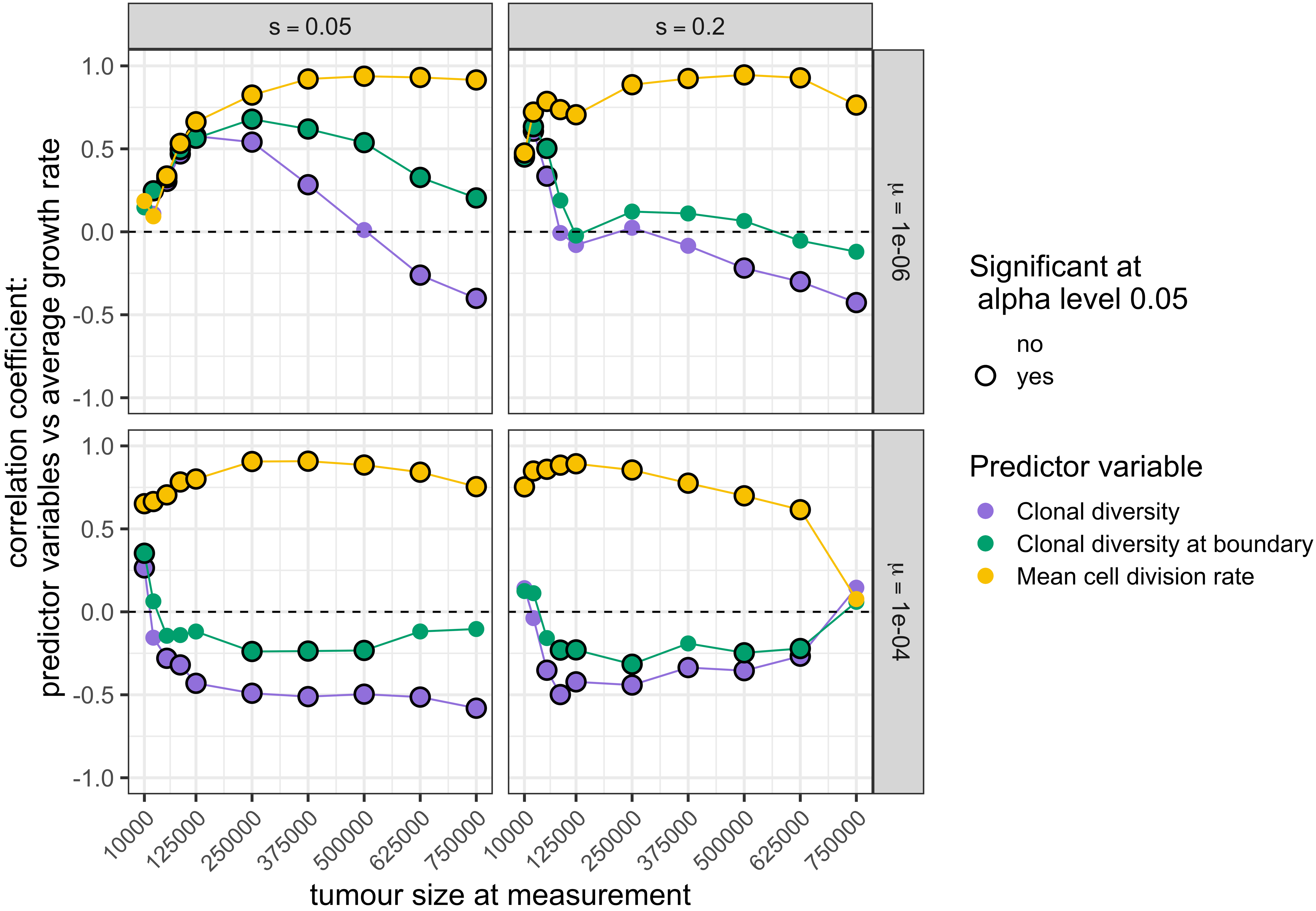
s = 0.05
s = 0.2
μ = 1e-06
μ = 1e-04
1.0
0.5
0.0
-0.5
-1.0
correlation coefficient:
predictor variables vs average growth rate
10000
125000
250000
375000
500000
625000
750000
tumour size at measurement
Significant at
alpha level 0.05
no
yes
Predictor variable
Clonal diversity
Clonal diversity at boundary
Mean cell division rate
