# Supplementary material for: When, why and how tumour clonal diversity predicts survival
Source: Evol Appl. 2020 Jul 18;13(7):1558–68. doi: 10.1111/eva.13057 (PMC7428820; doi:10.1111/eva.13057)
Supplement: Supplementary file 7 — Figure S7 [file EVA-13-1558-s007.pdf]

a

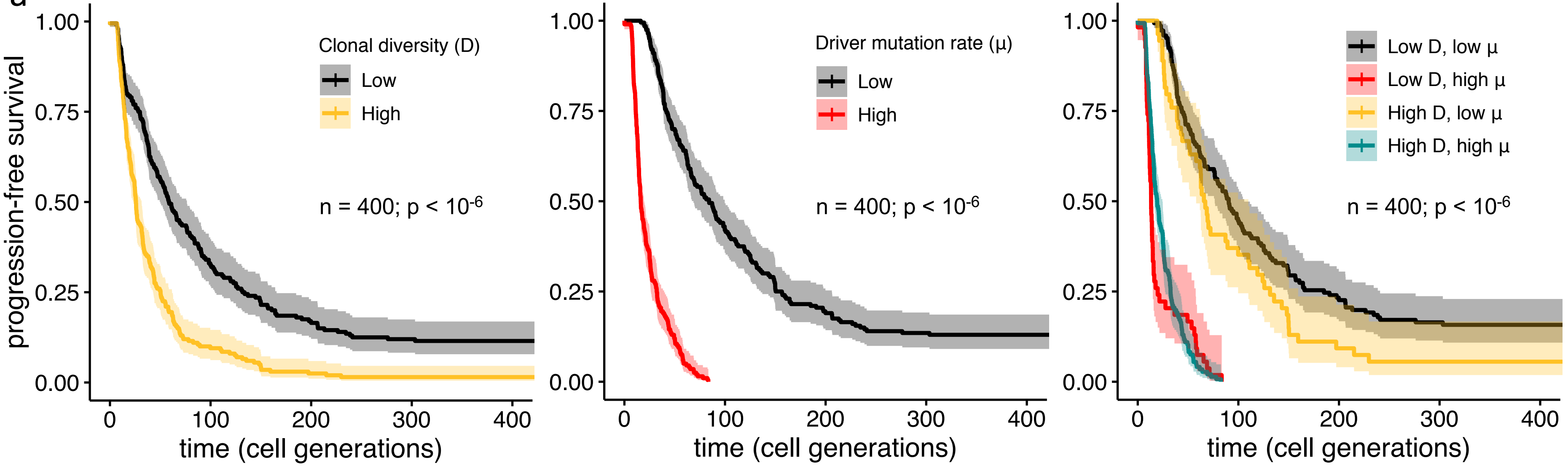

b

term	estimate	std. error	z	p. value
$\log_{10}(D)$	1.66	0.191	8.66	$<10^{-6}$

term	estimate	std. error	z	p. value
$\log_{10}(D)$	-7.78	1.15	-6.76	$<10^{-6}$
$\log_{10}(D) * \log_{10}(t + 10)$	5.89	1.07	5.49	$<10^{-6}$
$\log_{10}(\mu)$	-1.82	0.651	-2.8	0.00505
$\log_{10}(\mu) * \log_{10}(t + 10)$	4.06	0.699	5.81	$<10^{-6}$

term	estimate	std. error	z	p. value
$\log_{10}(D)$	0.0646	0.235	0.275	0.783
$\log_{10}(\mu)$	8.58	1.42	6.05	$<10^{-6}$
$\log_{10}(\mu) \times \log_{10}(t + 10)$	-1.88	0.789	-2.39	0.0171
$\log_{10}(b - 1)$	45	2.44	18.4	$<10^{-6}$
$\log_{10}(b - 1) \times \log_{10}(t + 10)$	-20.7	1.23	-16.9	$<10^{-6}$

Cox proportional hazards model
D = clonal diversity; t = time (cell generations); b = mean cell division rate; μ = driver mutation rate; s = mean driver mutation effect
Green rows contain terms with significant effects ($p < 0.05$)
